# ACSL3 is a promising therapeutic target for alleviating anxiety and depression in Alzheimer’s disease

**DOI:** 10.1007/s11357-024-01424-5

**Published:** 2024-11-13

**Authors:** Celeste Yin-Chieh Wu, Yulan Zhang, Peyton Howard, Fang Huang, Reggie Hui-Chao Lee

**Affiliations:** 1 Department of Neurology, Louisiana State University Health, LSU Health Sciences Center Shreveport, 1501 Kings Hwy, Shreveport, LA 71103-3932 USA; 2 Institute for Cerebrovascular and Neuroregeneration Research, Louisiana State University Health, Shreveport, LA USA; 3 Department of Cellular Biology and Anatomy, Louisiana State University Health, Shreveport, LA USA

**Keywords:** Alzheimer’s disease, Long-chain acyl-CoA synthetases, Depression, Anxiety, Brain-derived neurotrophic factor, Vascular endothelial growth factor C

## Abstract

Alzheimer’s disease (AD), the leading cause of dementia, affects over 55 million people worldwide and is often accompanied by depression and anxiety. Both significantly impact patients’ quality of life and impose substantial societal and economic burdens on healthcare systems. Identifying the complex regulatory mechanisms that contribute to the psychological and emotional deficits in AD will provide promising therapeutic targets. Biosynthesis of omega-3 (ω3) and omega-6 fatty acids (ω6-FA) through long-chain acyl-CoA synthetases (ACSL) is crucial for cell function and survival. This is due to ω3/6-FA’s imperative role in modulating the plasma membrane, energy production, and inflammation. While ACSL dysfunction is known to cause heart, liver, and kidney diseases, the role of ACSL in pathological conditions in the central nervous system (e.g., depression and anxiety) remains largely unexplored. The impact of ACSLs on AD-related depression and anxiety was investigated in a mouse model of Alzheimer’s disease (3xTg-AD). ACSL3 levels were significantly reduced in the hippocampus of aged 3xTg-AD mice (via capillary-based immunoassay). This reduction in ACAL3 was closely associated with increased depression and anxiety-like behavior (via forced swim, tail suspension, elevated plus maze, and sucrose preference test). Upregulation of ACSL3 via adenovirus in aged 3xTg-AD mice led to increased protein levels of brain-derived neurotrophic factor (BDNF) and vascular endothelial growth factor C (VEGF-C) (via brain histology, capillary-based immunoassay), resulting in alleviation of depression and anxiety symptoms. The present study highlights a novel neuroprotective role of ACSL3 in the brain. Targeting ACSL3 will offer an innovative approach for treating AD-related depression and anxiety.

**Supplementary Information:**

The online version contains supplementary material available at 10.1007/s11357-024-01424-5.

## Introduction

In the United States, an estimated 6.7 million Americans are living with Alzheimer’s disease (AD). This number is expected to reach 13.8 million by 2050, according to Alzheimer’s Association [1]. In addition to memory loss, AD patients often suffer neuro-psychological symptoms, including anxiety and depression. Growing evidence suggests that depression and anxiety accelerate the pathological progression of AD [2]. An experiment using AD mouse model has revealed a strong correlation between anxiety-like behavior and extensive formation of cerebral plaque and neurofibrillary tangles [3]. Medications to mitigate depression and anxiety have high therapeutic potential in the treatment of AD.

Although the accumulation of amyloid beta (Aβ) and phosphorylated tau proteins in the brain are the most prominent hallmark of AD, it remains debatable whether Aβ or phosphorylated tau is the primary cause of neurocognitive deficits and AD [4]. This debate is underscored by the fact that two FDA-approved anti-Aβ antibodies (Aducanumab and Lecanemab) have failed to reverse or cure AD [5–7]. Additionally, only a limited group of AD patients (17%) are eligible for anti-Aβ therapy due to high levels of Aβ [8, 9], highlighting the need to identify novel pathways leading to cell death and cognitive deficits in AD.

The brain is the second highest lipid content organ behind adipose tissues, with approximately 50% of its composition being lipids [10, 11]. Lipid homeostasis in the brain has received increasing attention in neurodegenerative disease research as lipids help maintain plasma membrane integrity and play critical roles in energy storage, signal transduction, protein anchoring [12]. Long-chain acyl-CoA synthetases (ACSLs) are essential enzymes in lipid homeostasis as they incorporate fatty acids into cellular phospholipids to control the biosynthesis of ω3- and ω6-fatty acids (FAs) [13–15]. ω3/6-FA deficiency triggered by downregulation of ACSL results in plasma membrane damage, inflammation, and energy failure [16–19]. Therefore, low ACSLs are closely related to heart, liver, and kidney diseases [13–15]. ACSLs’ role in neurodegenerative diseases (e.g., AD), however, has rarely been studied and remains largely unknown. Among the five different ACSL isoforms (1, 3, 4, 5, 6) [20], we chose to investigate ACSL3 because it is predominately expressed in the brain [15]. To elucidate the fundamental role of ACSL3 in AD-induced neuro-psychological deficits, we utilized a cell type-specific genetic approach [adeno-associated virus (AAV) overexpressing ACSL3 gene] in a mouse model of AD (aged 3xTg-AD mice). Our results suggest ACSL3 was downregulated in aged 3x-Tg-AD mice, while overexpression of ACSL3 via AAV increased neurotrophic factor levels [e.g., brain-derived neurotrophic factor (BDNF) and vascular endothelial growth factor C (VEGFC)], thereby alleviating depression- and anxiety-like behavior.

## Materials and methods

### Animals and study design

It is well-known that “Women who had given birth to five or more children were 70% more likely to develop AD than women who gave birth to fewer children” (according to the American Associate of Neurology 7/18/2018 press release). To model post-reproductive aging in mice, all experiments were conducted on young (1–3 months old) and aged (9–12 months old) female retired breeder 3x-Tg-AD mice [B6;129-Tg (APPSwe, tauP301L)1Lfa *Psen1*^*tm1Mpm*^/Mmjax, MMRC Strain #034830-JAX] [21]. To ensure the study was performed in an unbiased manner, all animals were purchased from Jackson Laboratory. All protocols for animal use were approved by the Animal Research Committee of Louisiana State University Health-Shreveport. To manipulate ACSL3 protein levels in the brain, mice received a single retro-orbital injection of AAV/PHP.eB-hSYN1-mACSL3-2A-eGFP (AAV-ACSL3, 1 × 10^11^ viral particles). All protein assay, brain histology, and animal behavioral studies were performed 30 days after AAV-ACSL3 treatment. In a separate set of experiments, we utilized ACSL3 activator (GW3965) as a proof-of-concept tool to support our AAV cell type-specific genetic approach. We administered GW3965 (Cayman Chemical, 20 mg/kg/day, IP) in young and aged 3xTg-AD female mice. Animal behavioral studies were performed 7 days post-treatment. The dosage of GW3965 (20 mg/kg/day) was derived from other *in vivo* mouse models of cerebral ischemia [22–24] and our preliminary studies (Supplementary Figure S2).

### RNA sequencing and bioinformatic analysis

Total RNA was isolated from 16 mouse brain samples (four young 3xTg, three aged 3xTg, and four aged 3xTg + AAV-ACSL3 mice) using RNeasy Mini kit (QIAGEN). The RNA was quantified with a Nanodrop ND-1000 spectrophotometer and then sent to the Novogene Corporation Inc. for sequencing. cDNA libraries were generated and quantified following manufacturer’s protocols (cDNA library concentration > 2 nM). The cDNA libraries were then sequenced using a HiSeq platform. Quality control was carried out and maintained with the Phred score. In RNA-seq analysis, gene expression levels were estimated by exon length and millions of mapped reads. Gene expression levels were normalized by calculating the Fragments Per Kilobase of transcript sequence per Million base pairs sequenced (FPKM) [25].

Differentially expressed (DE) genes were analyzed using Ingenuity Pathway Analysis (IPA, QIAGEN) to identify pathways, regulatory networks, and upstream regulators. Genes were shown as nodes, and the molecular relationship between two nodes were represented with either a solid (direct interactions) or a dashed (indirect interactions) line. IPA analyses whether the patterns of expression observed in the DE genes can be explained by the activation/inhibition of any of the considered regulators through a z-score calculation, *i.e.,* a statistical measure of the difference between the expected regulator-target relationship direction and the observed gene expression [26]. IPA was performed on a subset of DE genes, with a *P*-value ≤ 0.01 and a fold change (FC) ≥ 1.5.

### Capillary-based immunoassay via ProteinSimple^®^

Tissue Protein Extraction Reagent (T-PER®, Thermo Scientific, Cat#78510) and Halt™ Protease Inhibitor Cocktail (Halt™, Thermo Scientific, Cat#87785) were utilized to extract protein from the hippocampus and cortex. Tissue lysate was then diluted to the desired concentrations (1 µg/µl). The diluted protein samples were mixed with Fluorescent Master Mix (1:4) and heated at 95 °C for 5 min. Capillary electrophoresis coupled with a 12-230 kDa Separation Module (Wes, ProteinSimple®, San Jose, CA, USA) was used to quantify protein levels. The antibodies used included ACSL3 (1:50, NOVUS, NBP2-15252), IGF1R (1:20, Proteintech, 20254-1-AP), PDGFRβ (1:50, ThermoFisher, MA5-15143), and VEGFC (1:100, CUSABIO, CSB-PA07545A0Rb). The data were normalized to the total protein of the sample via the Total Protein Detection Module.

### Immunofluorescence staining

Upon completion of behavioral trials, the mice (*n* = 5 per group) were transcardially perfused with cold saline for 2 min, followed by 4% paraformaldehyde (PFA) for 5 min. Mice brain tissue was collected and stored at 4 °C in 4% PFA for 2 days. The tissue samples were then dehydrated with 30% sucrose solution for 7 days and embedded in Neg-50™ Frozen Section Medium (Fisher Scientific, Hampton, NH, USA). Tissue slicing was sectioned at a thickness of 25 μm, and 0.4% Triton X-100 was used to permeabilize the sections. To avoid non-specific binding of the antibodies, the permeabilized brain sections were incubated with 3% donkey serum for 45 min. Brain sections were later incubated with primary antibodies (1:300) at room temperature overnight. The antibodies used included ACSL3 (Proteintech, 20710-1-AP), NeuN (Millipore, MAB377), BDNF (NOVUS, NB100-98682), and VEGF-C (CSB-PA07545A0Rb, CUSABIO). The following day, the brain sections were washed with 0.1% Triton X-100 for 90 min and then incubated with corresponding secondary antibodies (1:500) (Invitrogen, A31573) for 1 h at room temperature in the dark. Fluorescent images were acquired with a Zeiss AxioObserver & Apotome microscope and Olympus CSU W1 Spinning Disk Confocal System. Results were counted from 2 sections per brain and were analyzed using ImageJ software (Version 1.50i; NIH; Bethesda, Maryland, USA).

### Enzyme-linked immunosorbent assay (ELISA)

100 µg of protein lysate from the hippocampus was extracted using T-PER® Tissue Protein Extraction Reagent (Thermo Scientific, Cat# 78510) and Halt™ Protease Inhibitor Cocktail (Thermo Scientific, Cat# 87785). Mature and pro-BDNF protein levels were measured by Mature BDNF/pro-BDNF Combo Rapid ELISA Kit (Bioscience, BEK-2211/2217, AUS). All steps were performed at room temperature. Samples were measured at 450 nm on a Sigma plate reader. Data was analyzed by four-parameter logistic curve fit.

### Forced swim test

The forced swim test is widely used to assess depression-like behavior in rodents. Mice were immersed in a transparent glass cylinder (18 cm height × 14 cm diameter) filled with water up to 15 cm. Animals were allowed to swim freely for 6 min. The total time the animals spent immobile was recorded. Immobility was defined as the animal remaining still, without visibly moving all four paws, for at least one second.

### Tail suspension test

The TST is another simple behavioral exam for assessment of depression-like behavior. Each mouse tail was fixed to suspend 50 ± 2 cm above the floor for 5 min. In the experiment, the immobility time of each mouse was manually recorded.

### Elevated plus maze

The apparatus consisted of an elevated cross-shaped maze with two opposite open arms and two enclosed arms, along with a central area. The maze is approximately 50 cm above the ground. In a dimly lit environment, the mice are placed at the center point of the maze, with their head facing the same direction. The mice were then allowed to explore the maze freely for 5 min. Anxiety-like behavior was recorded as the time spent on the two open arms and the number of entries into the open arms.

### Sucrose preference test

The sucrose preference test was used to evaluate the hedonic deficit, an indicator of anxiety, in mice. Prior to testing, the animals were housed individually and acclimated to two identical bottles filled with regular drinking water for 24 h. The drinking water in both bottles was then replaced with 2% sucrose water for 48 h. Upon completion of adaptation to the sucrose solution adaptation, the mice underwent a 12-h food and water deprivation. The mice were then provided one bottle of 2% sucrose water and one bottle of drinking water in their home cages. After 24 h, the two bottles were removed and weighed. Hedonic deficits in mice were evaluated via sucrose water consumption rate, calculated as follows: (consumption of sucrose water / total fluid consumption) X 100%.

### Statistical analysis

Results were expressed as means ± SD. The Shapiro-Wilk test in GraphPad Prism 9 was used to assess the normal distribution of the groups Statistical analysis was evaluated by one-way ANOVA (Tukey’s *post-hoc* test) using GraphPad Prism 9. *p* ≤0.05 was considered statistically significant.

## Results

### ACSL3 was significantly decreased in the hippocampus of aged 3xTg-AD female mice

**Fig. 1:**
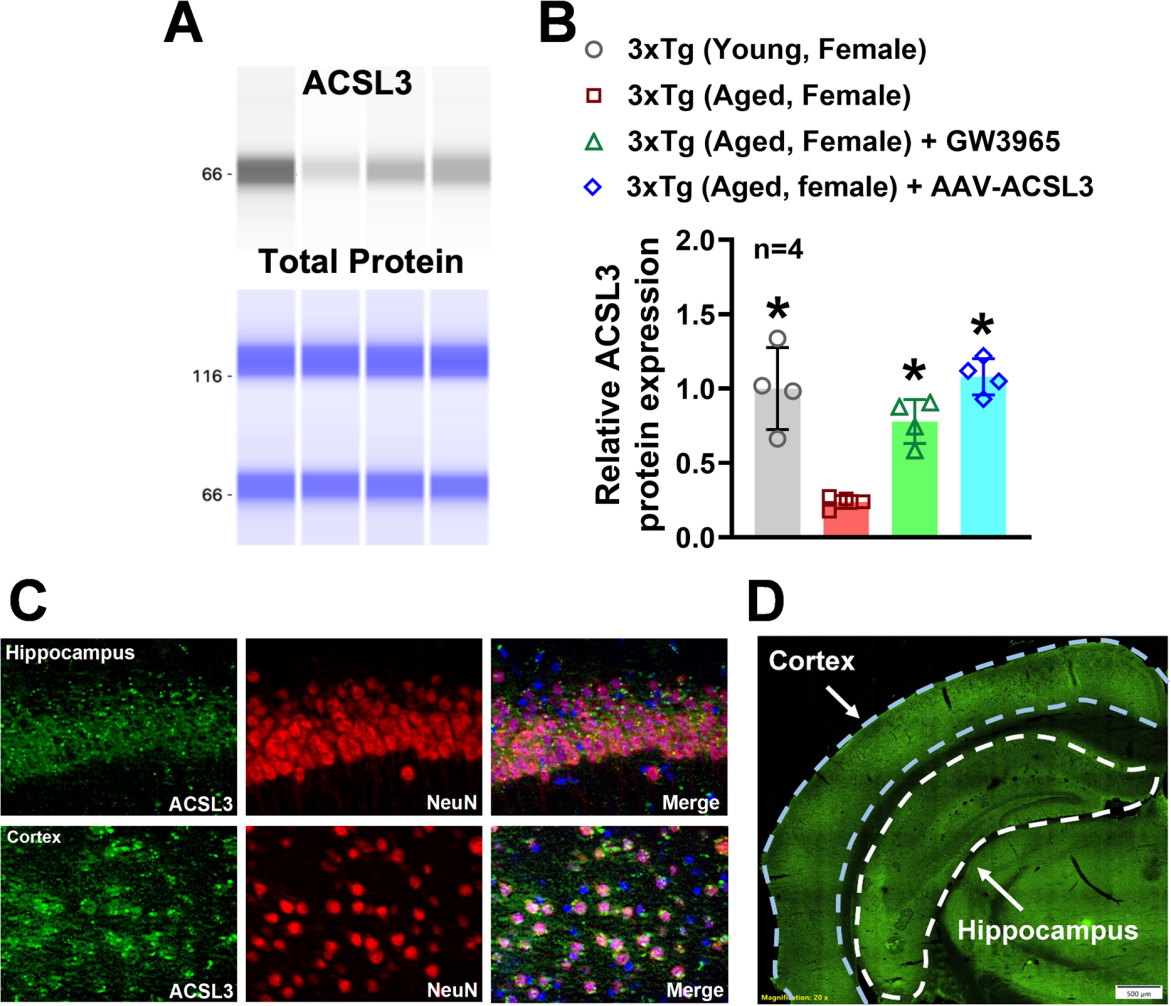
Expression of ACSL3 was significantly decreased in the brain of aged 3xTg-AD female mice, while treatment with AAV-ACSL3 or GW3965 increased ACSL3 protein levels (**A**) Relative protein levels of ACSL3 in the hippocampus of 3xTg-AD mice were measured by capillary-based immunoassay. ACSL3 bands at 66 kDa. Results were normalized with total protein and summarized in panel **B**. (**C**) Colocalization of ACSL3-immunoreactivity with NeuN in the mouse hippocampus and cortex. Neurons were stained for ACSL3 (green) and the pan-neuronal marker NeuN (red). Nuclei are counterstained with 4',6-diamidino-2-phenylindole (DAPI, blue). Scale bar = 100 μm. The vast majority of the neurons are positive for ACSL3. (**D**) GFP fluorescent imaging of a coronal brain section 30 days after a single retro-orbital injection of AAV-ACSL3. Scale bar = 500 µm. Green fluorescence indicates the presence of GFP-tagged AAV-ACSL3 particles. *p ≤ 0.05 indicates significantly different versus aged 3xTg-AD female mice, evaluated by one-way ANOVA with Tukey’s *post-hoc*

The ProteinSimple® capillary electrophoresis system was employed to measure relative protein levels of ACSL3 in the hippocampus. ACSL3 protein levels in the hippocampus of aged 3xTg-AD mice (0.23 ± 0.04) were significantly lower compared to those in young 3xTg-AD mice (1.00 ± 0.27) (Fig. 1A and B). Immunofluorescence staining was then utilized to identify specific cell types that express ACSL3 in the brain. Co-staining of NeuN and ACSL3 revealed high expression of ACSL3 in neurons within the hippocampus and cortex (Fig. 1C).

To overexpress ACSL3 specifically in neurons of aged 3xTg-AD mice, we used an AAV-PHP.eB vector expressing the ACSL3 gene under the synapsin promoter (AAV-ACSL3). Both fluorescent imaging of coronal brain sections and capillary electrophoresis confirmed the transduction efficiency of the AAV-PHP.eB. Thirty days after a single injection of AAV-ACSL3, hippocampal and cortical neurons with successful genetic transduction of ACSL6 showed high levels of green fluorescent protein (GFP) (Fig. 1D). Additionally, protein levels of ACSL3 in the hippocampus were significantly increased in aged 3xTg-AD mice treated with AAV-ACSL3 (1.08 ± 0.12) as compared to untreated aged 3xTg-AD mice (0.23 ± 0.04) (Fig. 1A and B). Our results suggest aged 3xTg-AD mice have dramatically low levels of ACSL3 in the brain, while AAV-ACSL3 treatment can effectively promote ACSL3 protein expression in aged 3xTg-AD mice.

### Overexpression of ACSL3 alleviates depression-like behavior in AD-mouse model

**Fig. 2:**
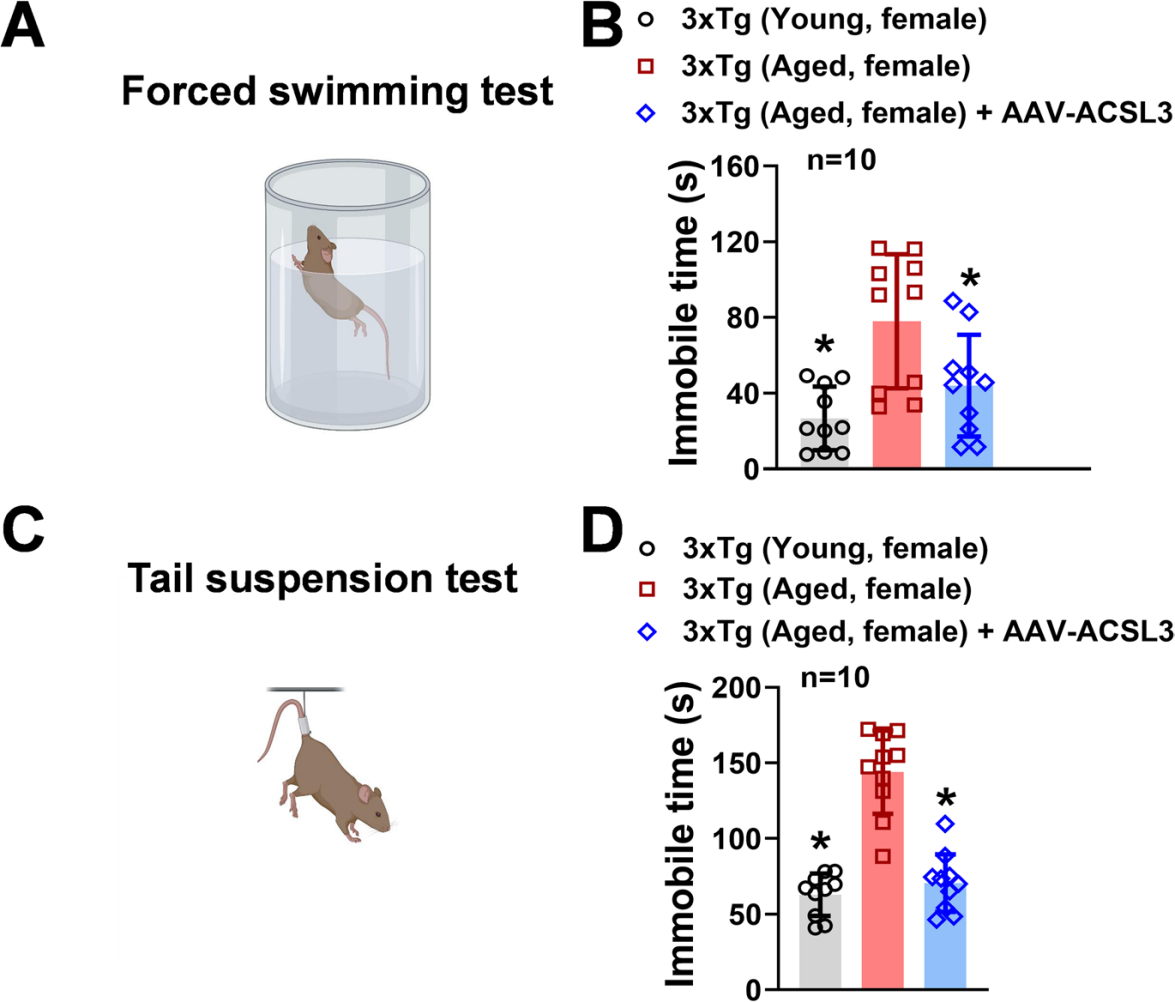
Overexpression of ACSL3 via AAV-ACSL3 alleviated depression-like behavior in 3xTg-AD mice. Aged female 3xTg-AD mice (9–12 months) received single retro-orbital injection of AAV-ACSL3 (1 × 10^11^ Viral particles). Forced swimming test (**A**) and tail suspension test (**B**) were implemented in mice 30 days after AAV-ACSL3 treatment. Results from forced swimming and tail suspension tests were summarized in panels **B** and **D**, respectively. *p ≤ 0.05 versus aged 3xTg-AD female mice, evaluated by one-way ANOVA with Tukey’s *post-hoc*

We conducted the forced swimming test to assess the impact of ACSL3 upregulation on depression-like symptoms in the AD-mouse model (Fig. 2). As shown in Fig. 2A and B, aged 3xTg-AD mice exhibited significantly longer immobility time (77.98 ± 35.38 s) compared to young 3xTg-AD mice (26.74 ± 16.75 s). The longer immobility time is an indicator of depression-like behavior. Interestingly, treatment of AAV-ACSL3 in aged 3xTg-AD mice significantly reduced immobility time (43.95 ± 26.85 s) (Fig. 2B). To further verify these findings, we employed tail suspension test as an independent approach to test whether upregulation of ACSL3 alleviates depression-like activity in AD-mouse model. Similar to the forced swimming test results, aged 3xTg-AD mice had significantly longer immobility time (143.95 ± 27.5 s) than young 3xTg-AD mice (62.95 ± 13.98 s) (Fig. 2C and D). With AAV-ACSL3 treatment, the immobility time in aged 3xTg-AD mice was significantly reduced (70.63 ± 19.03 s) suggesting alleviation of depression-like behavior.

### Overexpression of ACSL3 reduced anxiety-like behavior in 3xTg-AD mice

**Fig. 3:**
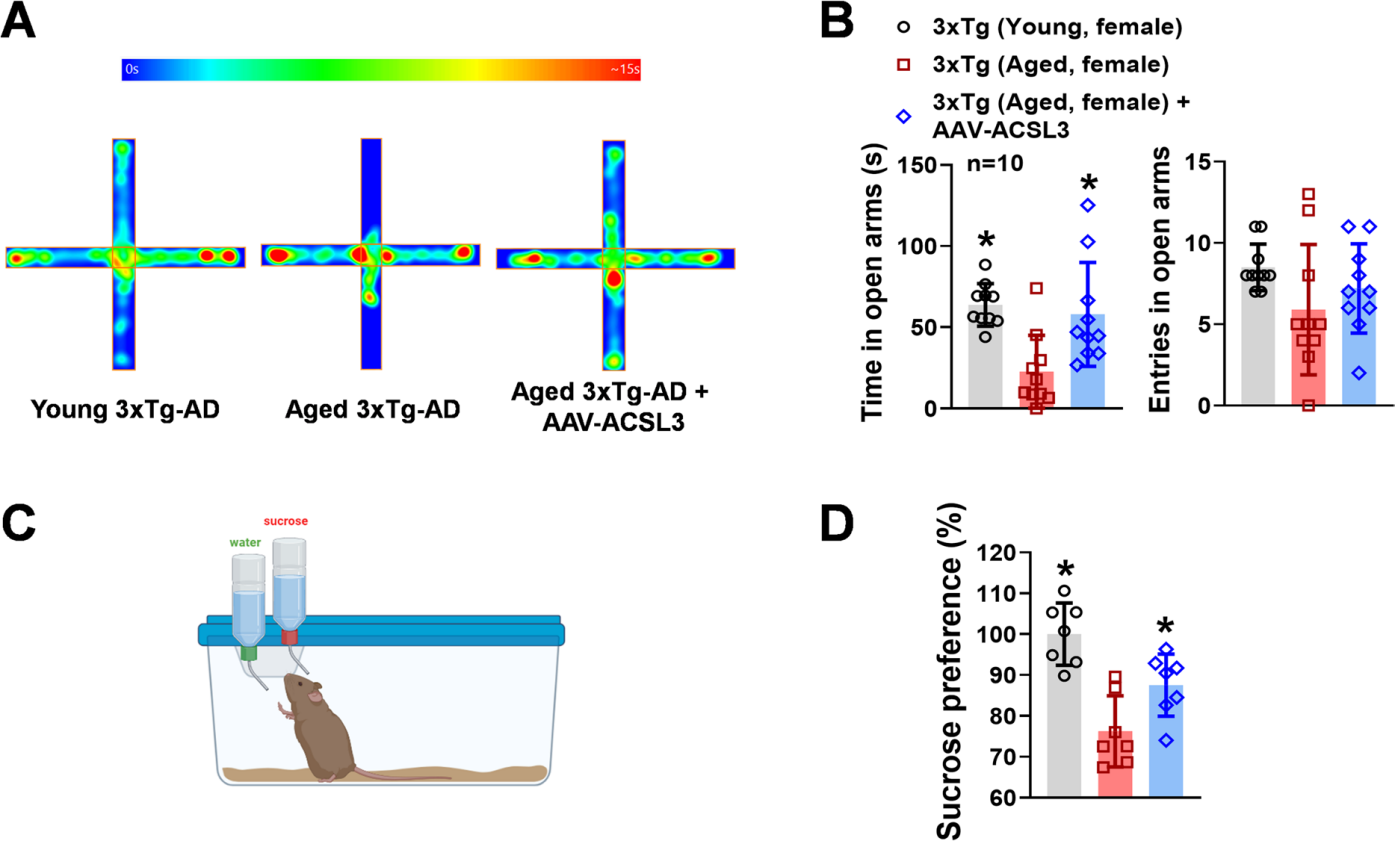
Anxiety-like behavior of aged 3xTg-AD mice was alleviated with AAV-ACSL3 treatment. The elevated plus maze (**A**) and sucrose preference test (**C**) were used to study the impact of ACSL3 on anxiety-like behavior in young (1–3 months old) and aged 3xTg-AD (9–12 months old) mice with and without AAV-ACSL3 treatment. Results from elevated plus maze and sucrose preference test were summarized in panels **B** and **D**. *p ≤ 0.05 versus aged 3xTg-AD female mice via one-way ANOVA with Tukey’s *post-hoc*

The prevalence of anxiety is also high in patients with AD [27, 28]. We used the elevated plus maze to evaluate the impact of ACSL3 on AD-related anxiety-like behavior in rodents. Although there was no significant difference in the number of entries into the open arms among the young 3xTg-AD, aged 3xTg-AD, and aged 3xTg-AD + AAV-ACSL3 (8.50 ± 1.43, 5.90 ± 4.01, 7.20 ± 2.74), aged 3xTg-AD mice spent significantly less time on the open arms (22.60 ± 22.47 s) than young 3xTg-AD mice (63.84 ± 13.09 s) (Fig. 3A and B). Treatment with AAV-ACSL3 significantly alleviated anxiety-like symptoms in aged 3xTg-AD mice, as evidenced by a significant increase in the time spent on the open arms (57.97 ± 32.00 s).

### Upregulation of ACSL3 treatment with AAV-ACSL3 reduced hedonic deficit in aged 3xTg-AD mice

One of the characteristic symptoms of depression is the inability to feel pleasure, known as anhedonia. We studied this symptom and the role of ACSL3 in hedonic deficit in the AD-mouse model via the sucrose preference test. As shown in Fig. 3C and D, the aged 3xTg-AD mice exhibited significantly decreased sucrose preference levels (76.23 ± 8.69%) as compared to the young 3xTg-AD mice (100 ± 7.60%). The low sucrose preference levels in aged 3xTg-AD mice indicated the presence of anhedonia [29, 30]. However, treatment with AAV-ACSL3 to enhance expression of ACSL3 in the hippocampus and cortex significantly ameliorated the hedonic deficit in aged 3xTg-AD mice (87.51 ± 7.60%).

### Upregulation of ACSL3 promoted BDNF and VEGF-C signaling pathways

The neurotrophic factors and their receptor signaling are closely involved in producing anti-depressant-like effects [31], with BDNF being the most well-known molecule in this context. We performed ELISA, capillary electrophoresis, and immunofluorescence to determine whether ACSL3 regulates neurotrophic factor levels. We first investigated the impact of ACSL3 on platelet-derived growth factor (PDGF) and insulin-like growth factor 1 (IGF1) signaling pathways by assessing their receptor levels in the hippocampus of the 3xTg-AD mice. Both pathways play critical roles in neuronal proliferation and survival [32–34]. Although our pilot study confirmed the expression of PDGF receptor beta (PDGFRβ) and IGF1 receptor (IGF1R) in the brain, we did not observe changes in PDGFRβ and IGF1R protein levels across control, AD, and AD + AAV-ACSL3 groups (Supplementary Figure S1). As a result, we shifted our focus to investigating the effects of reduced brain ACSL3 on BDNF and VEGF-C in the context of AD.

**Fig. 4:**
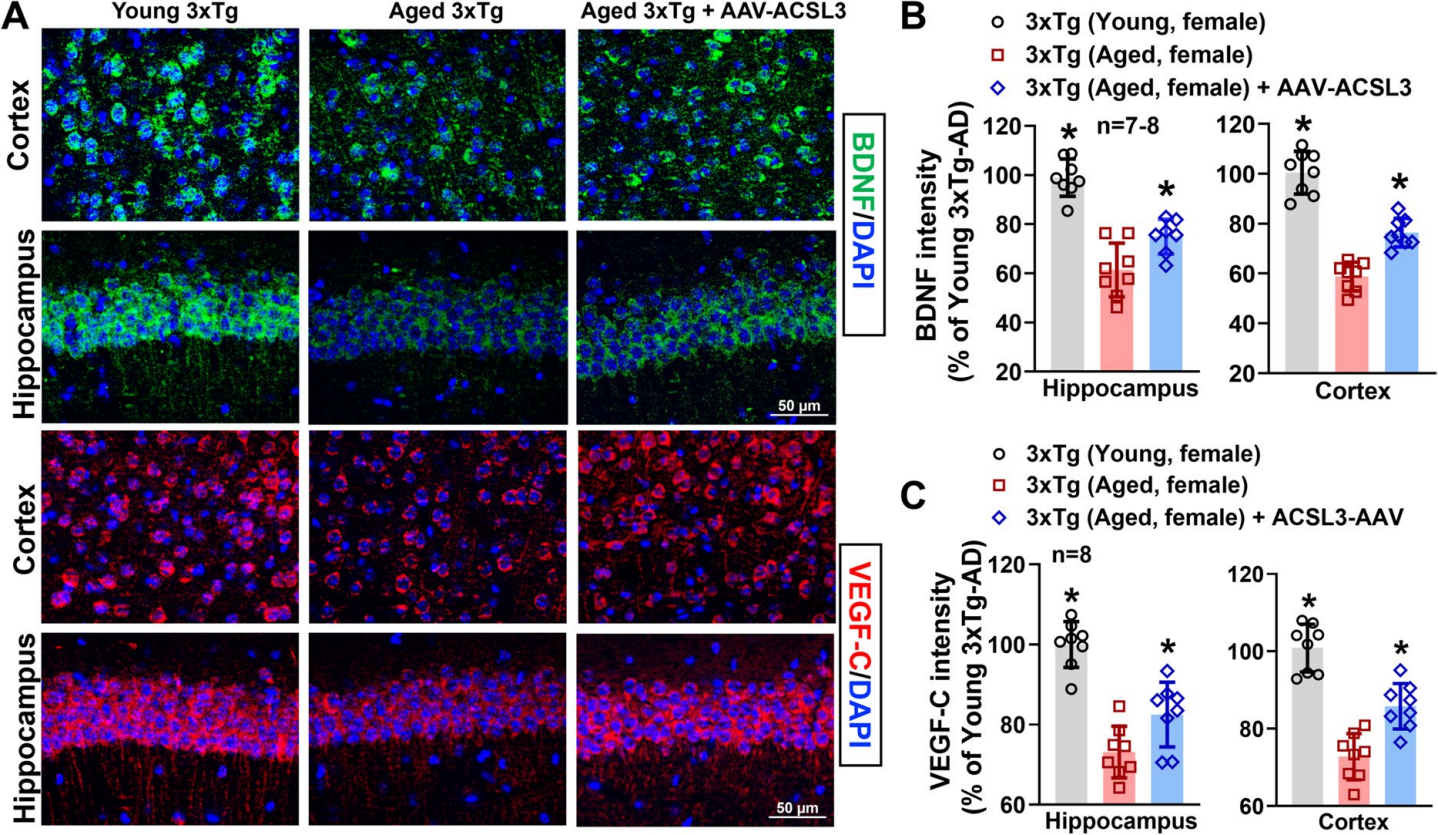
BDNF and VEGFc protein levels were reduced in aged 3xTg-AD mice but increased following AAV-ACSL3 treatment. (**A**) Representative immunofluorescence images of BDNF (green) and VEGF-C (red) in the CA1 region of the hippocampus and cortex. Quantification of the results were summarized in panels **B** and **C**. Scale bar = 50 µm. *p ≤ 0.05 versus aged 3xTg-AD female mice via one-way ANOVA with Tukey’s *post-hoc*

**Fig. 5:**
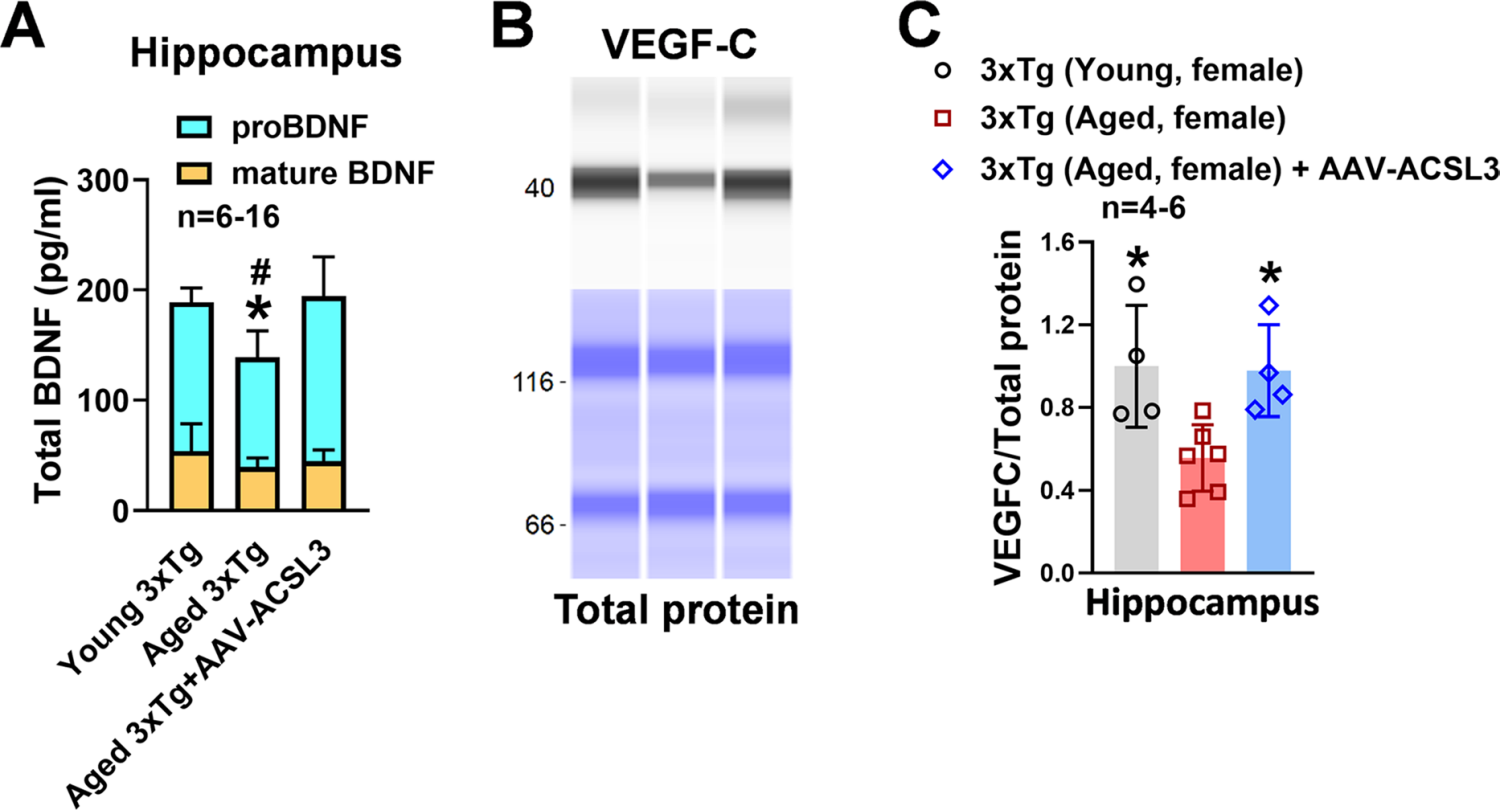
Overexpression of ACSL3 via AAV increased protein levels of proBDNF and VEGF-C in aged 3xTg-AD female mice. (**A**) Protein levels of pro and mature BDNF in the hippocampus of AD mice were measured via ELISA according to the manufacturer’s instructions. (**B**) Relative protein levels of VEGF-C in the hippocampus of 3xTg-AD mice were measured by capillary-based immunoassay. VEGF-C bands at 47 kDa. VEGF-C protein levels were normalized with total protein and summarized in panel **C**. *p ≤ 0.05 versus aged 3xTg-AD female mice via one-way ANOVA with Tukey’s *post-hoc*

Protein levels of BDNF in the hippocampus and cortex (98.98 ± 7.58 and 100.44 ± 8.59 vs 61.41 ± 10.93 and 58.76 ± 5.74) were significantly reduced in aged 3x-Tg mice as compared to young 3xTg mice. Upregulation of ACSL3 via AAV increased BDNF protein levels in aged 3xTg-AD mice (74.94 ± 7.07 and 76.41 ± 5.74) (Fig. 4A and B). Similarly, pro-BDNF levels in the hippocampal lysate were drastically reduced in aged 3xTg-AD mice as compared to young 3xTg-AD mice (99.11 ± 24.09 vs 134.64 ± 13.10) but increased with ACSL3-AAV treatment (149.37 ± 35.78) (Fig. 5A). Similar results were observed in the VEGF-C experiments. The fluorescence intensity of VEGF-C in the hippocampus and cortex was notably decreased in aged 3xTg-AD mice in comparison to young 3xTg mice. Aged AD mice treated with ACSL3-AAV exhibited higher VEGF-C fluorescence intensity in the hippocampus and cortex as compared to no treatment group (Fig. 4A and C) (100.00 ± 5.74 and 100.85 ± 6.18 vs 73.13 ± 6.43 and 72.75 ± 5.99). This observation was further confirmed by capillary electrophoresis, where protein levels of VEGF-C in the hippocampal lysate was significantly lower in aged 3xTg mice as compared to their young counterpart (0.48 ± 0.26 vs1.00 ± 0.29), while treatment with ACSL3-AAV increased VEGF-C protein levels (0.87 ± 0.30) (Fig. 5B and C).

**Fig. 6:**
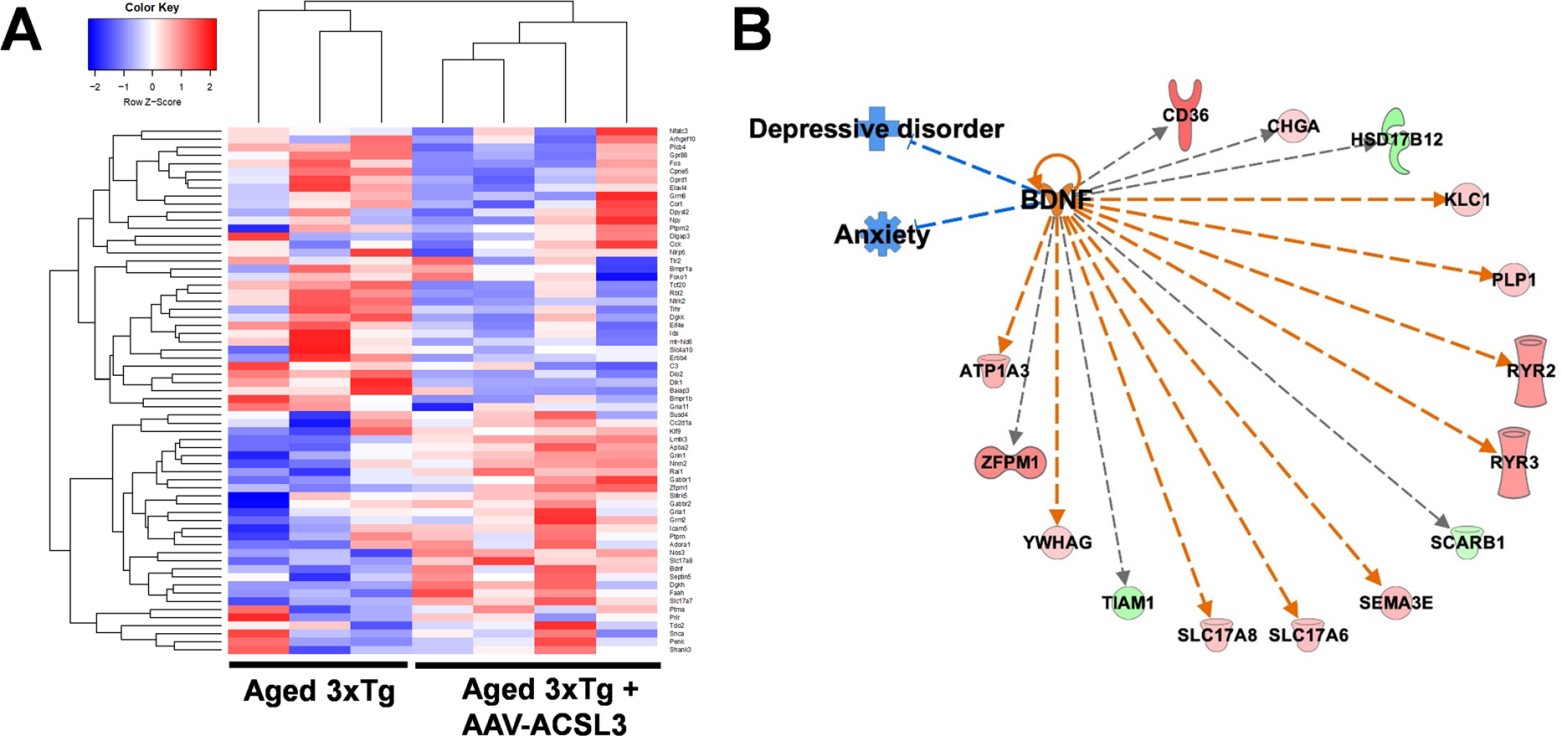
Gene expression profiling revealed that BDNF signaling pathways were involved in ACSL3-mediated anti-depression and anti-anxiety effects. RNA-sequencing of the mouse hippocampus was utilized to identify potential pathways involved ACSL3-mediated neuroprotection against AD. (**A**) Heatmap of 65 anxiety-related genes was generated from seven distinct mouse hippocampal tissues (three 3xTg-AD and four 3xTg-AD + AAV-ACSL3). (**B**) Differential gene expression was analyzed by Ingenuity Pathway Analysis (IPA). Genes shaded in green represent downregulated genes, while those in red are upregulated. Dashed lines indicate indirect interactions between genes. Blue and orange lines predict inhibition and activation, respectively. Grey lines indicate that the effects between two genes are not predicted based on the Ingenuity Knowledge Base

To confirm the mechanisms underlying ACSL3-mediated neuroprotection, we conducted RNA sequencing (Fig. 6A) and pathway-focused gene expression analysis (IPA) to identify potential signaling pathways involved. The results indicated the possible involvement of BDNF and its downstream genes in ACSL3-mediated antidepressant and anti-anxiety effects (Fig. 6B).

## Discussion

Neuro-psychological symptoms are prevalent among individuals with cognitive impairments and dementia. AD is particularly associated with these symptoms, as approximately 40% of AD patients exhibit depression and anxiety [27]. Psychological symptoms can occur even before the stage of cognitive impairment and memory loss [35–37]. Therefore, neuropsychological symptoms, such as depression and anxiety have served as early signs of AD [27]. Increasing evidence suggests that depression and anxiety can facilitate and even accelerate the pathological progression of AD [2]. Understanding the factors that contribute to mental disorders could lead to novel therapies and targets against AD.

ω3- and ω6-polyunsaturated fatty acids (PUFAs) are considered healthy polyunsaturated fatty acids (C18-C22). They contain more than one cis double bond and are found at high levels in fatty fish, eggs, flaxseeds, and nuts [38]. Over the past decade, ω3/6-PUFAs have drawn researcher’s attention in the area of neurodegenerative disease (e.g., brain tumor, stroke, and AD)^13^ due to their essential role in cell membrane homeostasis, anti-oxidative stress, and energy metabolism of the central nervous system. It is well known that many neurodegenerative diseases, including AD, are characterized by ω3/6-PUFAs deficiency. For example, levels of docosahexaenoic acid (DHA), an ω3-PUFA, in the mid-frontal and superior temporal cortex are often found to be lower in AD patients as compared to healthy individuals. AD patients typically have low plasma oleic acid and linoleic acid contents as well [39]. While the benefits of ω3/6-PUFAs consumption are well published, the use of ω3/6-PUFAs as therapeutics is not generally recommended because there is not enough evidence to determine if ω3/6-PUFA oral supplements can increase ω3/6-PUFAs levels in the brain. This is due to the fact that ω3/6-FAs enter the brain at fairly slow rates [40, 41]. The discrepancies in beneficial impacts of ω3/6-PUFA can also vary significantly depending on the type, dosage, and duration of intake [42, 43].

ω3/6-FAs transportation and metabolism in the brain heavily rely on ACSLs. These enzymes play a crucial role not only in the forming fatty acyl CoA for mitochondrial β-oxidation but also in acting as fatty acid transport proteins to facilitate the uptake of fatty acids across the plasma membrane [44–46]. ACSL6, in particular, has been reported to enrich DHA levels in the brain under normal physiological conditions through these roles [47–49]. Without ACSL6, there are significant consequences, such as a reduction in DHA-containing phospholipids [49], memory deficits, and increased neuroinflammation, highlighting the importance of ACSL6 in neuronal lipid metabolism [47].

Although ACSLs’ unique role in fatty acid transportation and metabolism has received increasing attention over the past decade, their function in the central nervous system, particularly in pathological conditions such as AD, remains largely unknown. Several studies suggested a significant upregulation of ACSL4 in stroke, cerebral ischemia, Parkinson’s disease, and multiple sclerosis [50–53]. Furthermore, ACSL4 promotes several cell death pathways, which include apoptosis, ferroptosis, and autophagy [54–56]. These studies indicate that the expression of ACSL4 in the brain is detrimental in neurodegenerative diseases.

In the present study, we are the first to observe a significant reduction of ACSL3 protein levels in the brains of aged 3xTg-AD mice, concomitant with depression- and anxiety-like behavior. Our findings led us to further delineate the physiological significance of ACSL3 in AD. Given that ACSL3 is highly expressed in neurons, as shown in Fig. 1C, we overexpressed ACSL3 in neurons of aged 3xTg-AD mice via AAV to study its role in the AD brain. We discovered that upregulation of ACSL3 in the neurons of aged 3xTg-AD mice attenuated depression- and anxiety-like behavior, as evidenced by the forced swim test, elevated plus maze, and sucrose preference test. Our research provides clear evidence that ACSL3, contrary to ACSL4, is beneficial in counteracting AD-related depression and anxiety.

The signaling axis of ACSL3 in AD remains largely unknown. To gain further insight into the cause of depression and anxiety as it relates to ACSL3, we analyzed differential gene and protein expression in aged 3xTg-AD mice treated with and without AAV-ACSL3. Results from RNA sequencing, Ingenuity Pathways Analysis (IPA), immunohistochemistry, and ELISA revealed that overexpression of ACSL3 via AAV drastically increased BDNF and VEGF-C levels in the hippocampus and cortex of aged 3xTg-AD mice, leading to better neuropsychological outcomes. To our knowledge, we are the first to establish this novel ACSL3/BDNF/VEGF-C axis in the context of AD.

BDNF, a neurotrophic factor, is crucial for promoting neuronal growth, survival, and synaptic plasticity [57]. Two pathophysiological hypotheses explaining BDNF’s beneficial impact against depression and anxiety have been proposed. First, BDNF acts as a neurotransmitter modulator (e.g., glutamate and N-methyl-D-aspartic acid) in the brain to enhance synaptic plasticity and synaptogenesis [58–61]. This hypothesis is supported by the high prevalence of synaptic dysfunction found in AD patients [62–64]. Abnormal synaptic transmission has been reported to be one of the critical factors causing depression and anxiety [65–69]. Second, according to anti-inflammatory theory, administration of BDNF suppresses the expression of various inflammatory cytokines, including tumor necrosis factor (TNF)-alpha, interleukin (IL)-1β, IL-6, and interferon (IFN)-alpha [70–73]. These inflammatory cytokines have been well-documented in the development of depression and anxiety [74–76].

On the other hand, VEGF-C was initially found to be associated with vascular development, vascular permeability, and angiogenesis. Recent studies suggest VEGF-C’s therapeutic potential in the treatment of several mood disorders such as depression, anxiety, schizophrenia and bipolar disorder [77]. VEGF-C serves as a regulator mediating postsynaptic function via N-methyl-D-aspartate type of glutamate receptors (GluNRs) in psychiatric disorders [78]. For instance, serum VEGF-C levels decline over time in patients with chronic depression [79], while intracisternal delivery of VEGF-C can alleviate stress-induced depression-like behavior in mice [80]. Although the molecular mechanisms underlying the antidepressant and anti-anxiety activities of VEGF-C are not fully understood, some studies suggest that VEGF exhibits immunosuppressive roles in T-cell function [81]. Others indicate that under ischemic conditions, VEGF-C stimulates microglia polarization from pro‐inflammatory (M1) to anti-inflammatory (M2) phenotype, leading to better neuronal survival and neurological outcomes [82].

## Limitations of the study

Male animals were not included in the study since women are more likely to develop a rapid progression of AD than men when predictive factors such as obesity, lifespan, and enhanced stroke severity are considered [83–88]. Additionally, over 15 studies have reported significantly greater Aβ accumulation in female 3xTg-AD mice v. their male counterparts between 3 and 23 months of age [89]. Further studies are needed to clarify the impact of ACSL3 on gender differences in AD.

From a therapeutic standpoint, we recognize that AAV-ACSL3 treatment may not be a practical option for AD patients. The pharmacological approach, such as specific agonist to activate ACSL3 signaling pathways, is currently limited by the absence of commercialized specific ACSL3 activator. However, recent studies suggest that activation of liver X receptor (LXR) via GW3965 can increase ACSL3 expression in the liver and human placental trophoblast cells [90, 91] through liver X receptor responsive element in the ACSL3 promoter [91]. It is important to note that GW3965 has been demonstrated to effectively cross blood-brain barrier [92–94], highlighting its potential as a therapeutic agent for AD.

We thus evaluated the therapeutic potential of GW3965 against depression- and anxiety-like behavior in AD. In this pilot study, IP injections of GW3965 (20 mg/kg/day for 7 days) significantly increased ACSL3 protein levels in the brain of 3xTg-AD mice (Fig. 1A and B). Treatment with GW3965 in AD mice provided robust neuroprotection alleviating depression- and anxiety-like behavior similarly to AAV-ACSL3 treatment (Supplementary Figure S2). These findings suggest that developing small-molecule activators of ACSL3 or utilizing GW3965 could be a novel therapeutic strategy for mitigating psychological disorders in AD patients. Further studies are needed to determine if a short-term seven-day treatment is sufficient to increase ACSL3 levels and whether this duration is adequate to change established pathology in the aged 3xTg mouse model, even after the treatment is discontinued.

## Conclusion

Through cell type-specific genetic (e.g., AAV-ACSL3) and pharmacological approaches (e.g., GW3965), we have demonstrated the neuroprotective role of ACSL3 in the AD mouse model. We observed significantly reduced ACSL3 protein levels in the brain of aged 3xTg-AD mice. Overexpression of ACSL3 via AAV markedly increased BDNF and VEGF-C levels in the hippocampus and cortex, which attenuated depression- and anxiety-like behavior. The use of GW3965 to activate ACSL3 signaling pathway could represent a novel therapeutic strategy for alleviating psychological disorders in AD patients.

## Supplementary Information

Below is the link to the electronic supplementary material.

Supplementary file1 (DOCX 879 KB)

## Data Availability

The datasets utilized during this study are available from the corresponding author upon request.

## References

[1] 2023 Alzheimer's disease facts and figures. Alzheimers Dement. 2023;19(4):1598-695.10.1002/alz.13016. 10.1002/alz.13016 36918389

[2] Dafsari FS, Jessen F. Depression-an underrecognized target for prevention of dementia in Alzheimer’s disease. Transl Psychiatry. 2020;10(1):160. 10.1038/s41398-020-0839-1. 32433512 10.1038/s41398-020-0839-1 PMC7239844

[3] Zhang YL, Xing RZ, Luo XB, Xu H, Chang RC, Zou LY, et al. Anxiety-like behavior and dysregulation of miR-34a in triple transgenic mice of Alzheimer’s disease. Eur Rev Med Pharmacol Sci. 2016;20(13):2853–62. 27424985

[4] Silvestro S, Valeri A, Mazzon E. Aducanumab and its effects on tau pathology: is this the turning point of amyloid hypothesis? Int J Mol Sci. 2022;23(4). 10.3390/ijms23042011. 10.3390/ijms23042011 PMC8880389 35216126

[5] Heidebrink JL, Paulson HL. Lessons learned from approval of aducanumab for Alzheimer’s disease. Annu Rev Med. 2024;75:99–111. 10.1146/annurev-med-051022-043645. 38285515 10.1146/annurev-med-051022-043645 PMC10926277

[6] Knopman DS, Jones DT, Greicius MD. Failure to demonstrate efficacy of aducanumab: an analysis of the EMERGE and ENGAGE trials as reported by Biogen, December 2019. Alzheimers Dement. 2021;17(4):696–701. 10.1002/alz.12213. 33135381 10.1002/alz.12213

[7] Cummings J, Apostolova L, Rabinovici GD, Atri A, Aisen P, Greenberg S, et al. Lecanemab: appropriate use recommendations. J Prev Alzheimers Dis. 2023;10(3):362–77. 10.14283/jpad.2023.30. 37357276 10.14283/jpad.2023.30 PMC10313141

[8] van der Kall LM, Truong T, Burnham SC, Dore V, Mulligan RS, Bozinovski S, et al. Association of beta-amyloid level, clinical progression, and longitudinal cognitive change in normal older individuals. Neurology. 2021;96(5):e662–70. 10.1212/WNL.0000000000011222. 33184233 10.1212/WNL.0000000000011222 PMC7884996

[9] Rosenberg A, Ohlund-Wistbacka U, Hall A, Bonnard A, Hagman G, Ryden M, et al. Beta-amyloid, tau, neurodegeneration classification and eligibility for anti-amyloid treatment in a memory clinic population. Neurology. 2022;99(19):e2102–13. 10.1212/WNL.0000000000201043. 36130840 10.1212/WNL.0000000000201043 PMC9651451

[10] Hamilton JA, Hillard CJ, Spector AA, Watkins PA. Brain uptake and utilization of fatty acids, lipids and lipoproteins: application to neurological disorders. J Mol Neurosci. 2007;33(1):2–11. 10.1007/s12031-007-0060-1. 17901539 10.1007/s12031-007-0060-1

[11] Yoon JH, Seo Y, Jo YS, Lee S, Cho E, Cazenave-Gassiot A, et al. Brain lipidomics: from functional landscape to clinical significance. Sci Adv. 2022;8(37):eadc9317. 10.1126/sciadv.adc9317. 36112688 10.1126/sciadv.adc9317 PMC9481132

[12] Yin F. Lipid metabolism and Alzheimer’s disease: clinical evidence, mechanistic link and therapeutic promise. FEBS J. 2023;290(6):1420–53. 10.1111/febs.16344. 34997690 10.1111/febs.16344 PMC9259766

[13] Dimas P, Montani L, Pereira JA, Moreno D, Trotzmuller M, Gerber J, et al. CNS myelination and remyelination depend on fatty acid synthesis by oligodendrocytes. Elife. 2019;8. 10.7554/eLife.44702. 10.7554/eLife.44702 PMC6504237 31063129

[14] Poitelon Y, Kopec AM, Belin S. Myelin fat facts: an overview of lipids and fatty acid metabolism. Cells. 2020;9(4). 10.3390/cells9040812. 10.3390/cells9040812 PMC7226731 32230947

[15] Yan S, Yang XF, Liu HL, Fu N, Ouyang Y, Qing K. Long-chain acyl-CoA synthetase in fatty acid metabolism involved in liver and other diseases: an update. World J Gastroenterol. 2015;21(12):3492–8. 10.3748/wjg.v21.i12.3492. 25834313 10.3748/wjg.v21.i12.3492 PMC4375570

[16] Bu SY, Mashek MT, Mashek DG. Suppression of long chain acyl-CoA synthetase 3 decreases hepatic de novo fatty acid synthesis through decreased transcriptional activity. J Biol Chem. 2009;284(44):30474–83. 10.1074/jbc.M109.036665. 19737935 10.1074/jbc.M109.036665 PMC2781602

[17] Saliakoura M, Reynoso-Moreno I, Pozzato C, Rossi Sebastiano M, Galie M, Gertsch J, Konstantinidou G. The ACSL3-LPIAT1 signaling drives prostaglandin synthesis in non-small cell lung cancer. Oncogene. 2020;39(14):2948–60. 10.1038/s41388-020-1196-5. 32034305 10.1038/s41388-020-1196-5 PMC7118021

[18] Padanad MS, Konstantinidou G, Venkateswaran N, Melegari M, Rindhe S, Mitsche M, et al. Fatty acid oxidation mediated by Acyl-CoA synthetase long chain 3 is required for mutant KRAS lung tumorigenesis. Cell Rep. 2016;16(6):1614–28. 10.1016/j.celrep.2016.07.009. 27477280 10.1016/j.celrep.2016.07.009 PMC4981512

[19] Magtanong L, Ko PJ, To M, Cao JY, Forcina GC, Tarangelo A, et al. Exogenous monounsaturated fatty acids promote a ferroptosis-resistant cell state. Cell Chem Biol. 2019;26(3):420-32 e9. 10.1016/j.chembiol.2018.11.016. 30686757 10.1016/j.chembiol.2018.11.016 PMC6430697

[20] Mashek DG, Li LO, Coleman RA. Long-chain acyl-CoA synthetases and fatty acid channeling. Future Lipidol. 2007;2(4):465–76. 10.2217/17460875.2.4.465. 20354580 10.2217/17460875.2.4.465 PMC2846691

[21] Tian S, Ye T, Cheng X. The behavioral, pathological and therapeutic features of the triple transgenic Alzheimer’s disease (3 x Tg-AD) mouse model strain. Exp Neurol. 2023;368:114505. 10.1016/j.expneurol.2023.114505. 37597764 10.1016/j.expneurol.2023.114505

[22] Naik SU, Wang X, Da Silva JS, Jaye M, Macphee CH, Reilly MP, et al. Pharmacological activation of liver X receptors promotes reverse cholesterol transport in vivo. Circulation. 2006;113(1):90–7. 10.1161/CIRCULATIONAHA.105.560177. 16365197 10.1161/CIRCULATIONAHA.105.560177

[23] Cui X, Chopp M, Zacharek A, Cui Y, Roberts C, Chen J. The neurorestorative benefit of GW3965 treatment of stroke in mice. Stroke. 2013;44(1):153–61. 10.1161/STROKEAHA.112.677682. 23204055 10.1161/STROKEAHA.112.677682 PMC3529962

[24] Cheng O, Ostrowski RP, Liu W, Zhang JH. Activation of liver X receptor reduces global ischemic brain injury by reduction of nuclear factor-kappaB. Neuroscience. 2010;166(4):1101–9. 10.1016/j.neuroscience.2010.01.024. 20096333 10.1016/j.neuroscience.2010.01.024 PMC2833222

[25] Trapnell C, Williams BA, Pertea G, Mortazavi A, Kwan G, van Baren MJ, et al. Transcript assembly and quantification by RNA-Seq reveals unannotated transcripts and isoform switching during cell differentiation. Nat Biotechnol. 2010;28(5):511–5. 10.1038/nbt.1621. 20436464 10.1038/nbt.1621 PMC3146043

[26] Kramer A, Green J, Pollard J Jr, Tugendreich S. Causal analysis approaches in ingenuity pathway analysis. Bioinformatics. 2014;30(4):523–30. 10.1093/bioinformatics/btt703. 24336805 10.1093/bioinformatics/btt703 PMC3928520

[27] Botto R, Callai N, Cermelli A, Causarano L, Rainero I. Anxiety and depression in Alzheimer’s disease: a systematic review of pathogenetic mechanisms and relation to cognitive decline. Neurol Sci. 2022;43(7):4107–24. 10.1007/s10072-022-06068-x. 35461471 10.1007/s10072-022-06068-x PMC9213384

[28] Mendez MF. The relationship between anxiety and Alzheimer’s disease. J Alzheimers Dis Rep. 2021;5(1):171–7. 10.3233/ADR-210294. 33981954 10.3233/ADR-210294 PMC8075566

[29] Verharen JPH, de Jong JW, Zhu Y, Lammel S. A computational analysis of mouse behavior in the sucrose preference test. Nat Commun. 2023;14(1):2419. 10.1038/s41467-023-38028-0. 37105954 10.1038/s41467-023-38028-0 PMC10140068

[30] Markov DD. Sucrose preference test as a measure of anhedonic behavior in a chronic unpredictable mild stress model of depression: outstanding issues. Brain Sci. 2022;12(10). 10.3390/brainsci12101287. 10.3390/brainsci12101287 PMC9599556 36291221

[31] Sathyanesan M, Newton SS. Antidepressant-like effects of trophic factor receptor signaling. Front Mol Neurosci. 2022;15:958797. 10.3389/fnmol.2022.958797. 36081576 10.3389/fnmol.2022.958797 PMC9445421

[32] Supeno NE, Pati S, Hadi RA, Ghani AR, Mustafa Z, Abdullah JM, et al. IGF-1 acts as controlling switch for long-term proliferation and maintenance of EGF/FGF-responsive striatal neural stem cells. Int J Med Sci. 2013;10(5):522–31. 10.7150/ijms.5325. 23532711 10.7150/ijms.5325 PMC3607237

[33] Wrigley S, Arafa D, Tropea D. Insulin-like growth factor 1: at the crossroads of brain development and aging. Front Cell Neurosci. 2017;11:14. 10.3389/fncel.2017.00014. 28203146 10.3389/fncel.2017.00014 PMC5285390

[34] Peng F, Dhillon NK, Yao H, Zhu X, Williams R, Buch S. Mechanisms of platelet-derived growth factor-mediated neuroprotection–implications in HIV dementia. Eur J Neurosci. 2008;28(7):1255–64. 10.1111/j.1460-9568.2008.06444.x. 18973553 10.1111/j.1460-9568.2008.06444.x PMC2716063

[35] Panza F, Frisardi V, Capurso C, D’Introno A, Colacicco AM, Imbimbo BP, et al. Late-life depression, mild cognitive impairment, and dementia: possible continuum? Am J Geriatr Psychiatry. 2010;18(2):98–116. 10.1097/JGP.0b013e3181b0fa13. 20104067 10.1097/JGP.0b013e3181b0fa13

[36] Gabryelewicz T, Styczynska M, Pfeffer A, Wasiak B, Barczak A, Luczywek E, et al. Prevalence of major and minor depression in elderly persons with mild cognitive impairment–MADRS factor analysis. Int J Geriatr Psychiatry. 2004;19(12):1168–72. 10.1002/gps.1235. 15526303 10.1002/gps.1235

[37] Donovan NJ, Locascio JJ, Marshall GA, Gatchel J, Hanseeuw BJ, Rentz DM, et al. Longitudinal association of amyloid beta and anxious-depressive symptoms in cognitively normal older adults. Am J Psychiatry. 2018;175(6):530–7. 10.1176/appi.ajp.2017.17040442. 29325447 10.1176/appi.ajp.2017.17040442 PMC5988933

[38] Abedi E, Sahari MA. Long-chain polyunsaturated fatty acid sources and evaluation of their nutritional and functional properties. Food Sci Nutr. 2014;2(5):443–63. 10.1002/fsn3.121. 25473503 10.1002/fsn3.121 PMC4237475

[39] Cunnane SC, Schneider JA, Tangney C, Tremblay-Mercier J, Fortier M, Bennett DA, Morris MC. Plasma and brain fatty acid profiles in mild cognitive impairment and Alzheimer’s disease. J Alzheimers Dis. 2012;29(3):691–7. 10.3233/JAD-2012-110629. 22466064 10.3233/JAD-2012-110629 PMC3409580

[40] Libinaki R, Gavin PD. Changes in bioavailability of Omega-3 (DHA) through Alpha-Tocopheryl Phosphate Mixture (TPM) after oral administration in rats. Nutrients. 2017;9(9). 10.3390/nu9091042. 10.3390/nu9091042 PMC5622802 28930161

[41] Destaillats F, Oliveira M, Bastic Schmid V, Masserey-Elmelegy I, Giuffrida F, Thakkar SK, et al. Comparison of the incorporation of DHA in circulatory and neural tissue when provided as triacylglycerol (TAG), monoacylglycerol (MAG) or phospholipids (PL) provides new insight into fatty acid bioavailability. Nutrients. 2018;10(5). 10.3390/nu10050620. 10.3390/nu10050620 PMC5986500 29762503

[42] Jiao J, Li Q, Chu J, Zeng W, Yang M, Zhu S. Effect of n-3 PUFA supplementation on cognitive function throughout the life span from infancy to old age: a systematic review and meta-analysis of randomized controlled trials. Am J Clin Nutr. 2014;100(6):1422–36. 10.3945/ajcn.114.095315. 25411277 10.3945/ajcn.114.095315

[43] Andrieu S, Guyonnet S, Coley N, Cantet C, Bonnefoy M, Bordes S, et al. Effect of long-term omega 3 polyunsaturated fatty acid supplementation with or without multidomain intervention on cognitive function in elderly adults with memory complaints (MAPT): a randomised, placebo-controlled trial. Lancet Neurol. 2017;16(5):377–89. 10.1016/S1474-4422(17)30040-6. 28359749 10.1016/S1474-4422(17)30040-6

[44] Black PN, Sandoval A, Arias-Barrau E, DiRusso CC. Targeting the fatty acid transport proteins (FATP) to understand the mechanisms linking fatty acid transport to metabolism. Immunol Endocr Metab Agents Med Chem. 2009;9(1):11–7. 10.2174/187152209788009850. 26635907 10.2174/187152209788009850 PMC4665979

[45] Black PN, DiRusso CC. Yeast acyl-CoA synthetases at the crossroads of fatty acid metabolism and regulation. Biochim Biophys Acta. 2007;1771(3):286–98. 10.1016/j.bbalip.2006.05.003. 16798075 10.1016/j.bbalip.2006.05.003

[46] Black PN, DiRusso CC. Vectorial acylation: linking fatty acid transport and activation to metabolic trafficking. Novartis Found Symp. 2007;286:127–38; discussion 38–41, 62–3, 96–203. 10.1002/9780470985571.ch11. 10.1002/9780470985571.ch11 18269179

[47] Fernandez RF, Pereyra AS, Diaz V, Wilson ES, Litwa KA, Martinez-Gardeazabal J, et al. Acyl-CoA synthetase 6 is required for brain docosahexaenoic acid retention and neuroprotection during aging. JCI Insight. 2021;6(11). 10.1172/jci.insight.144351. 10.1172/jci.insight.144351 PMC8262339 34100386

[48] Chouinard-Watkins R, Bazinet RP. ACSL6 is critical for maintaining brain DHA levels. Proc Natl Acad Sci USA. 2018;115(49):12343–5. 10.1073/pnas.1817557115. 30446610 10.1073/pnas.1817557115 PMC6298068

[49] Fernandez RF, Kim SQ, Zhao Y, Foguth RM, Weera MM, Counihan JL, et al. Acyl-CoA synthetase 6 enriches the neuroprotective omega-3 fatty acid DHA in the brain. Proc Natl Acad Sci USA. 2018;115(49):12525–30. 10.1073/pnas.1807958115. 30401738 10.1073/pnas.1807958115 PMC6298081

[50] Luoqian J, Yang W, Ding X, Tuo QZ, Xiang Z, Zheng Z, et al. Ferroptosis promotes T-cell activation-induced neurodegeneration in multiple sclerosis. Cell Mol Immunol. 2022;19(8):913–24. 10.1038/s41423-022-00883-0. 35676325 10.1038/s41423-022-00883-0 PMC9338013

[51] Bouchaoui H, Mahoney-Sanchez L, Garcon G, Berdeaux O, Alleman LY, Devos D, et al. ACSL4 and the lipoxygenases 15/15B are pivotal for ferroptosis induced by iron and PUFA dyshomeostasis in dopaminergic neurons. Free Radic Biol Med. 2023;195:145–57. 10.1016/j.freeradbiomed.2022.12.086. 36581060 10.1016/j.freeradbiomed.2022.12.086

[52] Tao WH, Shan XS, Zhang JX, Liu HY, Wang BY, Wei X, et al. Dexmedetomidine attenuates ferroptosis-mediated renal ischemia/reperfusion injury and inflammation by inhibiting ACSL4 via alpha2-AR. Front Pharmacol. 2022;13:782466. 10.3389/fphar.2022.782466. 35873574 10.3389/fphar.2022.782466 PMC9307125

[53] Tuo QZ, Liu Y, Xiang Z, Yan HF, Zou T, Shu Y, et al. Thrombin induces ACSL4-dependent ferroptosis during cerebral ischemia/reperfusion. Signal Transduct Target Ther. 2022;7(1):59. 10.1038/s41392-022-00917-z. 35197442 10.1038/s41392-022-00917-z PMC8866433

[54] Helfenberger KE, Argentino GF, Benzo Y, Herrera LM, Finocchietto P, Poderoso C. Angiotensin II regulates mitochondrial mTOR pathway activity dependent on Acyl-CoA synthetase 4 in adrenocortical cells. Endocrinology. 2022;163(12). 10.1210/endocr/bqac170. 10.1210/endocr/bqac170 36256598

[55] Lei G, Zhang Y, Koppula P, Liu X, Zhang J, Lin SH, et al. The role of ferroptosis in ionizing radiation-induced cell death and tumor suppression. Cell Res. 2020;30(2):146–62. 10.1038/s41422-019-0263-3. 31949285 10.1038/s41422-019-0263-3 PMC7015061

[56] Wu X, Zhi F, Lun W, Deng Q, Zhang W. Baicalin inhibits PDGF-BB-induced hepatic stellate cell proliferation, apoptosis, invasion, migration and activation via the miR-3595/ACSL4 axis. Int J Mol Med. 2018;41(4):1992–2002. 10.3892/ijmm.2018.3427. 29393361 10.3892/ijmm.2018.3427 PMC5810201

[57] Bathina S, Das UN. Brain-derived neurotrophic factor and its clinical implications. Arch Med Sci. 2015;11(6):1164–78. 10.5114/aoms.2015.56342. 26788077 10.5114/aoms.2015.56342 PMC4697050

[58] Kramar EA, Chen LY, Lauterborn JC, Simmons DA, Gall CM, Lynch G. BDNF upregulation rescues synaptic plasticity in middle-aged ovariectomized rats. Neurobiol Aging. 2012;33(4):708–19. 10.1016/j.neurobiolaging.2010.06.008. 20674095 10.1016/j.neurobiolaging.2010.06.008 PMC2978788

[59] Walz C, Jungling K, Lessmann V, Gottmann K. Presynaptic plasticity in an immature neocortical network requires NMDA receptor activation and BDNF release. J Neurophysiol. 2006;96(6):3512–6. 10.1152/jn.00018.2006. 17110740 10.1152/jn.00018.2006

[60] Miranda M, Morici JF, Zanoni MB, Bekinschtein P. Brain-derived neurotrophic factor: a key molecule for memory in the healthy and the pathological brain. Front Cell Neurosci. 2019;13:363. 10.3389/fncel.2019.00363. 31440144 10.3389/fncel.2019.00363 PMC6692714

[61] Marosi K, Mattson MP. BDNF mediates adaptive brain and body responses to energetic challenges. Trends Endocrinol Metab. 2014;25(2):89–98. 10.1016/j.tem.2013.10.006. 24361004 10.1016/j.tem.2013.10.006 PMC3915771

[62] Meftah S, Gan J. Alzheimer’s disease as a synaptopathy: evidence for dysfunction of synapses during disease progression. Front Synaptic Neurosci. 2023;15:1129036. 10.3389/fnsyn.2023.1129036. 36970154 10.3389/fnsyn.2023.1129036 PMC10033629

[63] Shankar GM, Walsh DM. Alzheimer’s disease: synaptic dysfunction and Abeta. Mol Neurodegener. 2009;4:48. 10.1186/1750-1326-4-48. 19930651 10.1186/1750-1326-4-48 PMC2788538

[64] Selkoe DJ. Alzheimer’s disease is a synaptic failure. Science. 2002;298(5594):789–91. 10.1126/science.1074069. 12399581 10.1126/science.1074069

[65] Duman RS, Aghajanian GK, Sanacora G, Krystal JH. Synaptic plasticity and depression: new insights from stress and rapid-acting antidepressants. Nat Med. 2016;22(3):238–49. 10.1038/nm.4050. 26937618 10.1038/nm.4050 PMC5405628

[66] Duman RS, Aghajanian GK. Synaptic dysfunction in depression: potential therapeutic targets. Science. 2012;338(6103):68–72. 10.1126/science.1222939. 23042884 10.1126/science.1222939 PMC4424898

[67] Yin JB, Liu HX, Shi W, Ding T, Hu HQ, Guo HW, et al. Various BDNF administrations attenuate SPS-induced anxiety-like behaviors. Neurosci Lett. 2022;788:136851. 10.1016/j.neulet.2022.136851. 36007708 10.1016/j.neulet.2022.136851

[68] Colucci-D'Amato L, Speranza L, Volpicelli F. Neurotrophic factor BDNF, physiological functions and therapeutic potential in depression, neurodegeneration and brain cancer. Int J Mol Sci. 2020;21(20). 10.3390/ijms21207777. 10.3390/ijms21207777 PMC7589016 33096634

[69] Castren E, Monteggia LM. Brain-derived neurotrophic factor signaling in depression and antidepressant action. Biol Psychiatry. 2021;90(2):128–36. 10.1016/j.biopsych.2021.05.008. 34053675 10.1016/j.biopsych.2021.05.008

[70] Capuron L, Miller AH. Cytokines and psychopathology: lessons from interferon-alpha. Biol Psychiatry. 2004;56(11):819–24. 10.1016/j.biopsych.2004.02.009. 15576057 10.1016/j.biopsych.2004.02.009

[71] Carniel BP, da Rocha NS. Brain-derived neurotrophic factor (BDNF) and inflammatory markers: perspectives for the management of depression. Prog Neuropsychopharmacol Biol Psychiatry. 2021;108:110151. 10.1016/j.pnpbp.2020.110151. 33096156 10.1016/j.pnpbp.2020.110151

[72] Xu D, Lian D, Wu J, Liu Y, Zhu M, Sun J, et al. Brain-derived neurotrophic factor reduces inflammation and hippocampal apoptosis in experimental Streptococcus pneumoniae meningitis. J Neuroinflammation. 2017;14(1):156. 10.1186/s12974-017-0930-6. 28778220 10.1186/s12974-017-0930-6 PMC5545027

[73] Porter GA, O’Connor JC. Brain-derived neurotrophic factor and inflammation in depression: pathogenic partners in crime? World J Psychiatry. 2022;12(1):77–97. 10.5498/wjp.v12.i1.77. 35111580 10.5498/wjp.v12.i1.77 PMC8783167

[74] Felger JC, Lotrich FE. Inflammatory cytokines in depression: neurobiological mechanisms and therapeutic implications. Neuroscience. 2013;246:199–229. 10.1016/j.neuroscience.2013.04.060. 23644052 10.1016/j.neuroscience.2013.04.060 PMC3741070

[75] Jeon SW, Kim YK. Neuroinflammation and cytokine abnormality in major depression: cause or consequence in that illness? World J Psychiatry. 2016;6(3):283–93. 10.5498/wjp.v6.i3.283. 27679767 10.5498/wjp.v6.i3.283 PMC5031928

[76] Lima Giacobbo B, Doorduin J, Klein HC, Dierckx R, Bromberg E, de Vries EFJ. Brain-derived neurotrophic factor in brain disorders: focus on neuroinflammation. Mol Neurobiol. 2019;56(5):3295–312. 10.1007/s12035-018-1283-6. 30117106 10.1007/s12035-018-1283-6 PMC6476855

[77] Pu J, Liu Y, Gui S, Tian L, Xu S, Song X, et al. Vascular endothelial growth factor in major depressive disorder, schizophrenia, and bipolar disorder: a network meta-analysis. Psychiatry Res. 2020;292:113319. 10.1016/j.psychres.2020.113319. 32717712 10.1016/j.psychres.2020.113319

[78] De Rossi P, Harde E, Dupuis JP, Martin L, Chounlamountri N, Bardin M, et al. A critical role for VEGF and VEGFR2 in NMDA receptor synaptic function and fear-related behavior. Mol Psychiatry. 2016;21(12):1768–80. 10.1038/mp.2015.195. 26728568 10.1038/mp.2015.195 PMC5116482

[79] Pisoni A, Strawbridge R, Hodsoll J, Powell TR, Breen G, Hatch S, et al. Growth factor proteins and treatment-resistant depression: a place on the path to precision. Front Psychiatry. 2018;9:386. 10.3389/fpsyt.2018.00386. 30190686 10.3389/fpsyt.2018.00386 PMC6115516

[80] Dai W, Yang M, Xia P, Xiao C, Huang S, Zhang Z, et al. A functional role of meningeal lymphatics in sex difference of stress susceptibility in mice. Nat Commun. 2022;13(1):4825. 10.1038/s41467-022-32556-x. 35974004 10.1038/s41467-022-32556-x PMC9381547

[81] Ribatti D. Immunosuppressive effects of vascular endothelial growth factor. Oncol Lett. 2022;24(4):369. 10.3892/ol.2022.13489. 36238855 10.3892/ol.2022.13489 PMC9494354

[82] Ju S, Xu C, Wang G, Zhang L. VEGF-C induces alternative activation of microglia to promote recovery from traumatic brain injury. J Alzheimers Dis. 2019;68(4):1687–97. 10.3233/JAD-190063. 30958378 10.3233/JAD-190063

[83] Vina J, Lloret A. Why women have more Alzheimer’s disease than men: gender and mitochondrial toxicity of amyloid-beta peptide. J Alzheimers Dis. 2010;20(Suppl 2):S527–33. 10.3233/JAD-2010-100501. 20442496 10.3233/JAD-2010-100501

[84] Prince M, Ali GC, Guerchet M, Prina AM, Albanese E, Wu YT. Recent global trends in the prevalence and incidence of dementia, and survival with dementia. Alzheimers Res Ther. 2016;8(1):23. 10.1186/s13195-016-0188-8. 27473681 10.1186/s13195-016-0188-8 PMC4967299

[85] Nebel RA, Aggarwal NT, Barnes LL, Gallagher A, Goldstein JM, Kantarci K, et al. Understanding the impact of sex and gender in Alzheimer’s disease: a call to action. Alzheimers Dement. 2018;14(9):1171–83. 10.1016/j.jalz.2018.04.008. 29907423 10.1016/j.jalz.2018.04.008 PMC6400070

[86] Appelros P, Stegmayr B, Terent A. Sex differences in stroke epidemiology: a systematic review. Stroke. 2009;40(4):1082–90. 10.1161/STROKEAHA.108.540781. 19211488 10.1161/STROKEAHA.108.540781

[87] Dufouil C, Seshadri S, Chene G. Cardiovascular risk profile in women and dementia. J Alzheimers Dis. 2014;42(Suppl 4):S353–63. 10.3233/JAD-141629. 25351109 10.3233/JAD-141629

[88] Glader EL, Stegmayr B, Norrving B, Terent A, Hulter-Asberg K, Wester PO, et al. Sex differences in management and outcome after stroke: a Swedish national perspective. Stroke. 2003;34(8):1970–5. 10.1161/01.STR.0000083534.81284.C5. 12855818 10.1161/01.STR.0000083534.81284.C5

[89] Dennison JL, Ricciardi NR, Lohse I, Volmar CH, Wahlestedt C. Sexual dimorphism in the 3xTg-AD mouse model and its impact on pre-clinical research. J Alzheimers Dis. 2021;80(1):41–52. 10.3233/JAD-201014. 33459720 10.3233/JAD-201014 PMC8075398

[90] Dong B, Kan CF, Singh AB, Liu J. High-fructose diet downregulates long-chain acyl-CoA synthetase 3 expression in liver of hamsters via impairing LXR/RXR signaling pathway. J Lipid Res. 2013;54(5):1241–54. 10.1194/jlr.M032599. 23427282 10.1194/jlr.M032599 PMC3622321

[91] Weedon-Fekjaer MS, Dalen KT, Solaas K, Staff AC, Duttaroy AK, Nebb HI. Activation of LXR increases acyl-CoA synthetase activity through direct regulation of ACSL3 in human placental trophoblast cells. J Lipid Res. 2010;51(7):1886–96. 10.1194/jlr.M004978. 20219900 10.1194/jlr.M004978 PMC2882745

[92] Namjoshi DR, Martin G, Donkin J, Wilkinson A, Stukas S, Fan J, et al. The liver X receptor agonist GW3965 improves recovery from mild repetitive traumatic brain injury in mice partly through apolipoprotein E. PLoS ONE. 2013;8(1):e53529. 10.1371/journal.pone.0053529. 23349715 10.1371/journal.pone.0053529 PMC3547922

[93] Donkin JJ, Stukas S, Hirsch-Reinshagen V, Namjoshi D, Wilkinson A, May S, et al. ATP-binding cassette transporter A1 mediates the beneficial effects of the liver X receptor agonist GW3965 on object recognition memory and amyloid burden in amyloid precursor protein/presenilin 1 mice. J Biol Chem. 2010;285(44):34144–54. 10.1074/jbc.M110.108100. 20739291 10.1074/jbc.M110.108100 PMC2962513

[94] Fitz NF, Cronican A, Pham T, Fogg A, Fauq AH, Chapman R, et al. Liver X receptor agonist treatment ameliorates amyloid pathology and memory deficits caused by high-fat diet in APP23 mice. J Neurosci. 2010;30(20):6862–72. 10.1523/JNEUROSCI.1051-10.2010. 20484628 10.1523/JNEUROSCI.1051-10.2010 PMC2883862

