# Supplementary material for: ACSL3 is a promising therapeutic target for alleviating anxiety and depression in Alzheimer’s disease
Source: GeroScience. 2024 Nov 13;47(2):2383–97. doi: 10.1007/s11357-024-01424-5 (PMC11978576; doi:10.1007/s11357-024-01424-5)
Supplement: Supplementary file 1 — Supplementary file1 (DOCX 879 KB) [file 11357_2024_1424_MOESM1_ESM.docx]

**
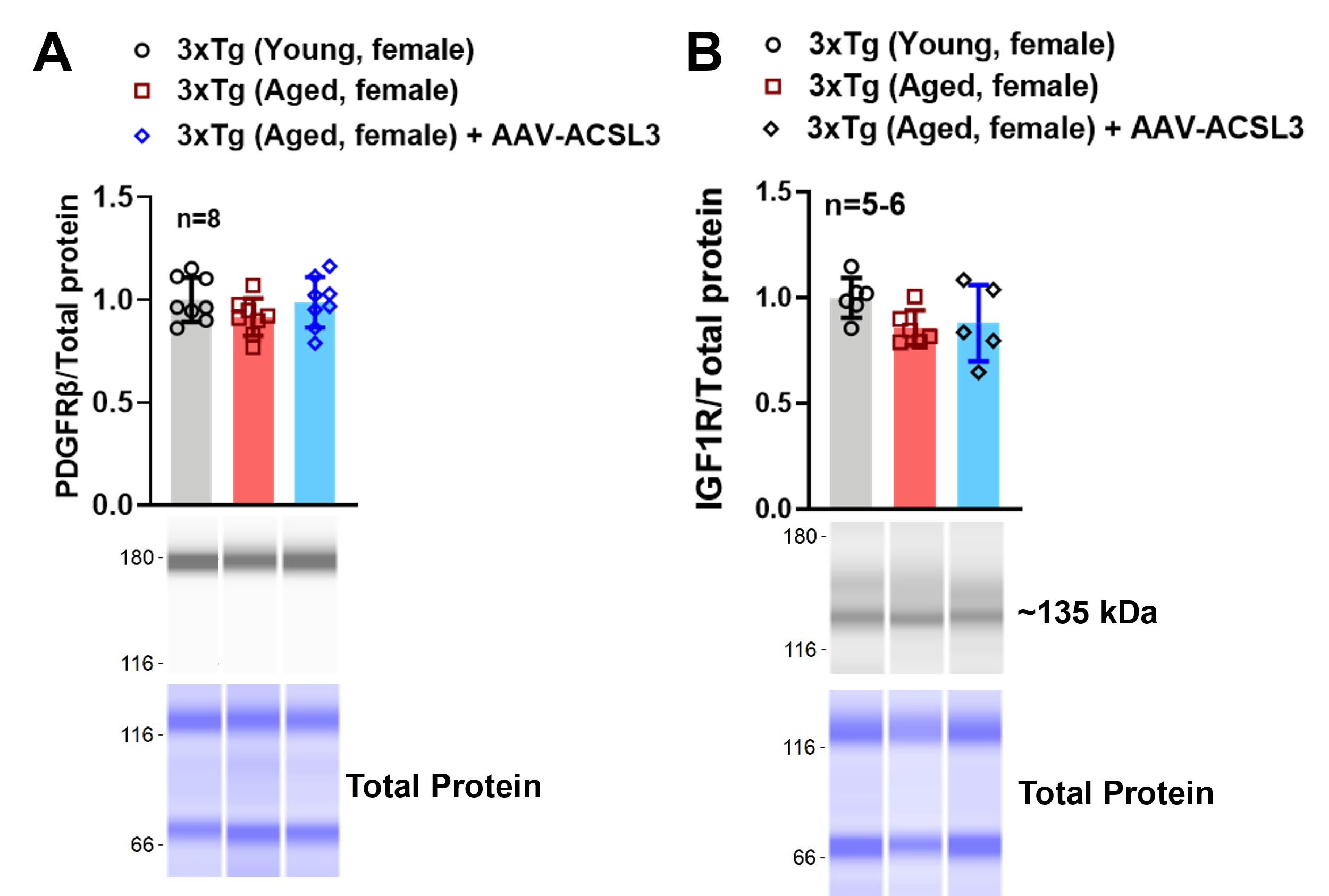
**

**Figure S1. ACSL3 overexpression via AAV had no effect on PDGFRβ and IGF1R protein levels in aged 3xTg-AD female mice.** Protein levels of PDGFRβ **(A)** and IGF1R **(B)** in the hippocampus of AD mice were measured via capillary-based immunoassay. PDGFRβ and IGF1R protein levels were normalized to total protein and presented as bar graphs. Results were analyzed via one-way ANOVA with Tukey’s *post-hoc*.

**
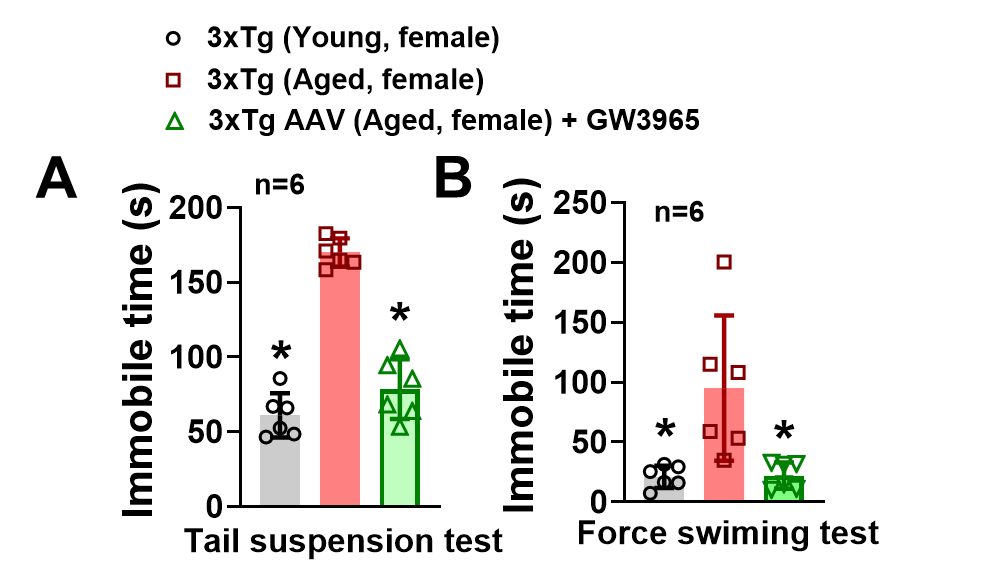
**

**Figure S2. ACSL3 upregulation exhibits antidepressant-like effects in 3xTg-AD mice.** Aged female 3xTg-AD mice (9-12 months) were treated with GW3965 (20mg/kg/day, ip). Behavioral paradigms were implemented in mice 7 days after GW3965 treatment. GW3965 observably reduced the immobility time of aged 3xTg-AD mice in TST and FST. *p≤0.05 indicates overall significantly different from aged 3xTg-AD mice, evaluated by one-way ANOVA with Tukey’s *post-hoc*.
